# Effects of *n*−3 Polyunsaturated Fatty Acids on Cardiac Ion Channels

**DOI:** 10.3389/fphys.2012.00245

**Published:** 2012-07-09

**Authors:** Cristina Moreno, Álvaro Macías, Ángela Prieto, Alicia de la Cruz, Teresa González, Carmen Valenzuela

**Affiliations:** ^1^Instituto de Investigaciones Biomédicas “Alberto Sols” (CSIC-UAM)Madrid, Spain

**Keywords:** *n*−3 PUFAs, atrial fibrillation, arrhythmias, heart failure

## Abstract

Dietary *n*−3 polyunsaturated fatty acids (PUFAs) have been reported to exhibit antiarrhythmic properties, and these effects have been attributed to their capability to modulate ion channels. In the present review, we will focus on the effects of PUFAs on a cardiac sodium channel (Na_v_1.5) and two potassium channels involved in cardiac atrial and ventricular repolarization (K_v_) (K_v_1.5 and K_v_11.1). *n*−3 PUFAs of marine (docosahexaenoic, DHA and eicosapentaenoic acid, EPA) and plant origin (alpha-linolenic acid, ALA) block K_v_1.5 and K_v_11.1 channels at physiological concentrations. Moreover, DHA and EPA decrease the expression levels of K_v_1.5, whereas ALA does not. DHA and EPA also decrease the magnitude of the currents elicited by the activation of Na_v_1.5 and calcium channels. These effects on sodium and calcium channels should theoretically shorten the cardiac action potential duration (APD), whereas the blocking actions of *n*−3 PUFAs on K_v_ channels would be expected to produce a lengthening of cardiac action potential. Indeed, the effects of *n*−3 PUFAs on the cardiac APD and, therefore, on cardiac arrhythmias vary depending on the method of application, the animal model, and the underlying cardiac pathology.

## Introduction

**Figure 1:**
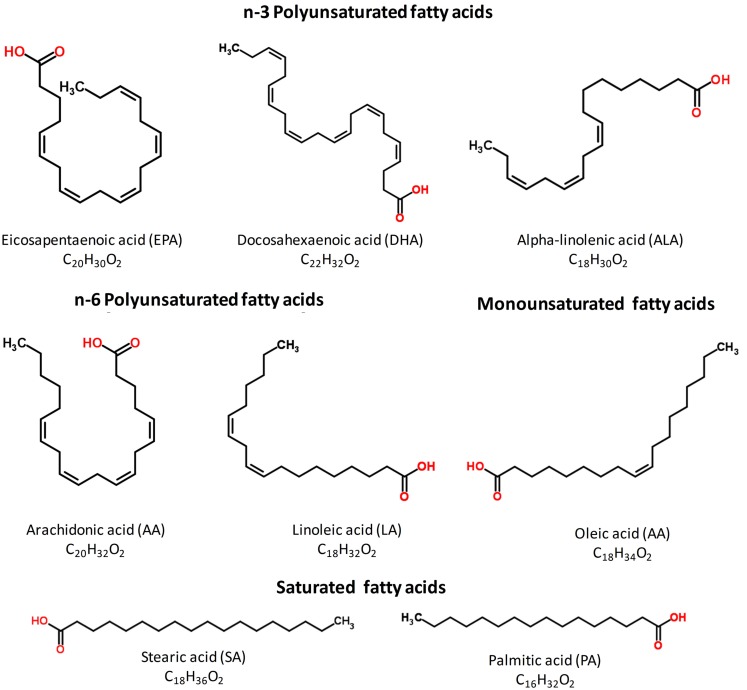
**Structure of the main *n***− **3 (EPA, DHA, and ALA), *n***− **6 (AA and LA), monounsaturated (OA), and saturated (SA and PA) PUFAs present in the human diet**. Taken from the free ChemSpider Database (http://www.chemspider.com/).

Omega-3 (*n*−3) polyunsaturated fatty acids (PUFAs) are essential nutrients that must be acquired from the diet and that are required for normal development and cellular function. *n*−3 PUFAs are derived from two sources: (1) ALA (18:3 *n*−3), which is found in vegetable oils (such as flaxseed, canola, and soybean oils) and walnuts, and (2) EPA (20:5 *n*−3) and DHA (22:6 *n*−3), which are found in oily fish, fish oil, and seafood (Figure 1). After the Industrial Revolution, the dramatic increase in the *n*−6-to-*n*−3 ratio in the diets of the populations of Western countries has, at least in part, contributed to the rise in cardiovascular disease (CVD; De Caterina et al., 2003; Leaf et al., 2003). Sinclair et al. described the rarity of CVD in Greenland Eskimos, who consumed a diet rich in *n*−3 PUFAs (whale, seal, and fish; Sinclair, 1953, 1956). Since that time, a large amount of evidence from cellular, animal studies (Billman et al., 1997, 1999; Leifert et al., 2000), and from clinical trial outcomes (Burr et al., 1989; GISSI-Prevenzione Investigators, 1999; Tanaka et al., 2008; Tavazzi et al., 2008) has suggested that an increased intake of fish oil fatty acids has favorable effects on cardiovascular health. Analyses of these trials have concluded that these beneficial effects mainly occur through the prevention of sudden cardiac death (SCD), which is often preceded by ventricular arrhythmias, indicating that *n*−3 PUFAs are antiarrhythmic (GISSI-Prevenzione Investigators, 1999; Marchioli et al., 2002). However, not all studies have demonstrated the cardioprotective effects on CVD of PUFA consumption. Proarrhythmic actions have been described for *n*−3 PUFAs in animal models during acute regional myocardial ischemia (Coronel et al., 2007). Moreover, the recent Alpha OMEGA and OMEGA randomized trials, involving patients who had suffered a myocardial infarction, did not show any improvement in the clinical results following *n*−3 PUFAs supplementation (Kromhout et al., 2010; Rauch et al., 2010), and a deleterious effect due to an increased risk of cardiac death was reported in men with stable angina (without myocardial infarction) who were advised to eat fish (Burr et al., 2003), and in patients with implantable cardioverter defibrillators (ICDs; Raitt et al., 2005). These differences could be explained by the fact that a diet rich in fish oil could be pro- or antiarrhythmic depending on the underlying arrhythmogenic mechanism.

**Figure 2:**
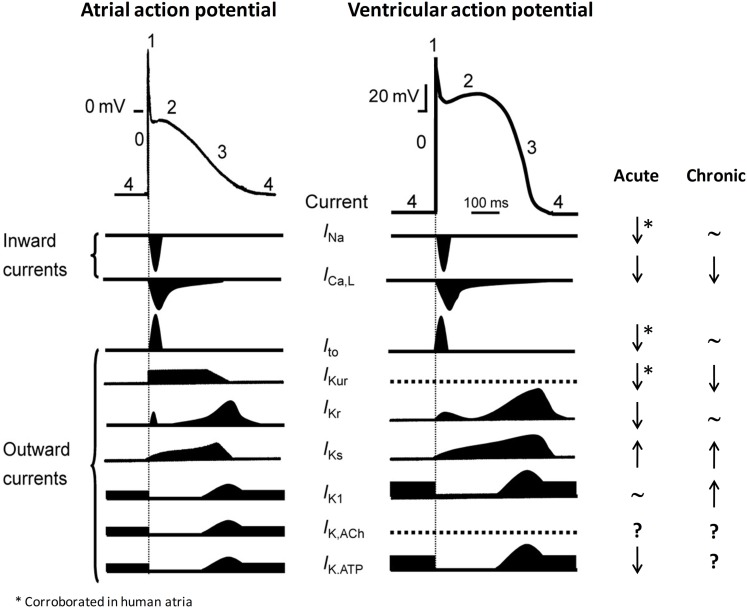
**Cardiac AP, ionic currents involved in its genesis and maintenance, and the effects of *n***− **3 PUFAs on each current under acute and chronic conditions**. ↑, Increase; ↓, decrease; or ∼, no changes in the current amplitude. *Studied in human atria. ?, Not tested. With permission from Ravens and Cerbai (2008).

In any case, the mechanism underlying the anti- or pro-arrhythmic effect after *n*−3 supplementation is thought to be related to the modulation of the cardiac ion channels involved in the genesis and/or maintenance of cardiac action potentials (APs). *n*−3 PUFAs inhibit the fast sodium current (*I*_Na_), ultrafast activating delayed outward potassium current (*I*_Kur_), transient outward potassium current (*I*_to_), rapidly activating delayed rectifying outward potassium current (*I*_Kr_) L-type calcium inward current (*I*_Ca_), and Na^+^-Ca^2+^ exchange current (*I*_NCX_), and enhanced slowly activating delayed rectifying outward potassium current (*I*_Ks_) and inward rectifying potassium current (*I*_K1_; Honoré et al., 1994; Xiao et al., 1995, 1997; Doolan et al., 2002; Jude et al., 2003; Guizy et al., 2005, 2008; Verkerk et al., 2006; Dujardin et al., 2008). Because the configuration and duration of the cardiac AP are highly relevant for arrhythmogenesis, in the present review, we summarize the acute and chronic effects of *n*−3 PUFAs on the *I*_Na_, *I*_Kur_, and *I*_Kr_ (Figure 2) and we will relate these effects with the pro- and antiarrhythmic effects on different animal models of arrhythmia, as well as to the results observed in the clinical trials.

## Effects of *n*−3 PUFAs on Sodium Channels

The fast inward cardiac sodium current is responsible for the genesis of the AP (phase 0) in most cells of excitable tissues and plays a pivotal role in impulse conduction. Thus, the ability of agents to modulate *I*_Na_ may constitute an important factor to prevent, terminate, or exacerbate cardiac arrhythmias (Echt et al., 1991).

In 1995, it was reported that EPA reduced membrane electrical excitability of heart myocytes, increased the threshold for the genesis of the cardiac AP and hyperpolarized the resting membrane potential (Kang et al., 1995). As it was expected, when the effects of EPA were studied on the fast sodium current in isolated neonatal rat cardiac myocytes (Xiao et al., 1995) or in the current generated after activation of cloned human Na_v_1.5 expressed in a mammalian cell line (Xiao et al., 1998), an inhibition of the *I*_Na_ occurred. These effects on sodium channels were concentration-, time-, and voltage-dependent with an IC_50_ of 4.8 and 0.5 μM respectively. These IC_50_ values are within the *n*−3 PUFAs physiological plasma range in the human being (Burtis et al., 1999). EPA shifted the inactivation curve toward more negative potentials by ∼20 mV whereas the activation kinetics of the channel was unaffected. EPA slightly hyperpolarized the resting membrane potential (likely due to an enhancement of *I*_K1_) and made more positive the threshold for the activation of Na_v_1.5 channel. In addition, the transition from the rested to the inactivated state was markedly accelerated in the presence of EPA but the recovery from inactivation was only slightly prolonged (Xiao et al., 1998; Dujardin et al., 2008). This effect could account for the greater relative refractory period-action potential duration (APD) ratio. Other *n*−3 (DHA and ALA), *n*−6 PUFAs (AA), and monounsaturated fatty acid (oleic acid, OA) reduced *I*_Na_ being the *n*−3 PUFAs the more potent ones.

In adult rat cardiomyocytes, sodium current was also inhibited by EPA, DHA, and ALA, DHA being the most potent *n*−3 PUFA blocking sodium channels (IC_50_ of 6 μM), followed by EPA (IC_50_ of 16 μM) and ALA (IC_50_ of 27 μM). Similarly to that observed in *I*_Na_ recorded in neonatal cardiomyocytes; DHA, EPA, and ALA shifted the voltage dependence of inactivation toward more negative potentials (∼20–23 mV). However, and in contrast to the effects on the sodium channels of neonatal cardiomyocytes, these three PUFAs shifted the voltage dependence of activation toward more positive potentials (∼8–12 mV; Leifert et al., 1999). In contrast with the acute effects, peak *I*_Na_ was unaffected by incorporated *n*−3 PUFAs in ventricular myocytes isolated from pigs and rats that were fed a diet rich in fish oil (Leifert et al., 2000; Verkerk et al., 2006). In both studies voltage dependence of activation remained unaltered, whereas a shift (<8 mV) in the inactivation toward more negative potentials was observed.

It is not clear how PUFAs modulate the activity of ion channels, if they modify the current through indirect effects on the lipid bilayer or if they directly interact with the channel protein. The dominant view is that *n*−3 PUFAs directly interact with the protein of the ion channel. A very useful technique used to establish an interacting site is to introduce single directed point mutations in the wild type protein and to determine whether the substitution modifies the expected action of the ligand. Due to the striking similarities between the *n*−3 PUFAs effects and those induced by local anesthetics, the effects of *n*−3 PUFAs were analyzed on Na_v_1.5 channels carrying point amino acid mutations that have been shown to modify the action of local anesthetics (Xiao et al., 2001). Interestingly, the substitution of an asparagine by a lysine at position 406 (N406K) at the segment 6 of the domain 1 of Na_v_1.5 channel significantly reduced the potency of EPA to inhibit *I*_Na_, mimicking bupivacaine effects and suggesting a direct interaction with this protein (Nau et al., 2000; Xiao et al., 2001). Also, the effects of *n*−3 PUFAs on the sodium current generated by the activation of Na_v_1.5 channels expressed in HEK293 cells were modified by the cotransfection with accessory β subunits (Xiao et al., 2000).

On the basis of these results, sodium channel blockade produced by *n*−3 PUFAs is responsible, at least in part, for the antiarrhythmic or proarrhythmic effects observed in clinical trials. In the clinics, class I antiarrhythmic drugs, such as flecainide, are useful in the treatment of atrial fibrillation (Fuster et al., 2011). Thus, we can speculate that the sodium current inhibition properties of *n*−3 PUFAs can account for the beneficial effects observed in clinical trials focused on atrial fibrillation (Calo et al., 2005). Conversely, blockade of the cardiac sodium channel can be deleterious under certain pathological conditions in which arrhythmias are based on reentrant activity, such as during acute myocardial ischemia. Sodium channel blockade by acute administration of *n*−3 PUFAs, likely facilitates reentry by slowing conduction velocity and incorporated *n*−3 PUFAs shorten APD and effective refractory period (ERP), because these conditions favor a reentrant circuit to arise. In fact, an increase in the risk of cardiac events has been reported in patients with acute ischemia (e.g., angina pectoris) and in patients with histories of sustained ventricular tachycardia or ventricular fibrillation, as well as arrhythmias triggered by reentry (Burr et al., 2003; Brouwer et al., 2009).

## Effects of *n*−3 PUFAs on Repolarizing Currents

The ultrarapid activated outward potassium current is the native counterpart to K_v_1.5 channels in human atria and contribute to the repolarization process of the atrial AP (Fedida et al., 1993; Snyders et al., 1993). Since the early nineties, it is known that K_v_1.5 current is inhibited by the acute exposition to *n*−3 (DHA), and *n*−6 PUFAs (AA), but not by monounsaturated (OA) and saturated fatty acids (palmitic acid, PA and stearic acid, SA) in stably expressing K_v_1.5 mammalian cells (Honoré et al., 1994). The inhibition was time- and concentration-dependent and occurred at physiological concentrations (IC_50_ for AA and DHA ∼20–30 μM) only when these PUFAs were applied from the external side of the membrane. Both *n*−6 and *n*−3 fatty acid shifted the activation curve toward more negative potentials ∼16 mV and accelerated the activation process. All these results are indicative of an open channel mechanism of block. The same characteristic block was induced by DHA and AA on the *I*_Kur_ current in mouse neonatal cardiomyocytes, in rat embryonic ventricular cells (Honoré et al., 1994) and, more interestingly, in human atrial myocytes (IC_50_ for EPA and DHA 17.5 and 4.3 μM, respectively; Li et al., 2009).

Guizy et al. (2008) compared acute versus chronic effects of the plant derived *n*−3 PUFA ALA on the inhibition of K_v_1.5 channels. The acute ALA blocking properties resembles to those previously described by Honoré et al. (1994) for DHA and AA. However, in the case of ALA, the K_v_1.5 inhibition was also voltage-dependent. The chronic effects on the channel function and on its protein expression levels were assessed by incubating the cells with ALA 10 μM for 24 and 48 h. The effects of ALA on the K_v_1.5 current were similar to those obtained when the cells were acutely exposed to ALA. EPA and DHA, but not ALA, reduced the steady-state levels of K_v_1.5 protein in a concentration-dependent manner at concentrations higher than 30 μM. However, it has also described that chronic treatment with EPA at low concentrations (∼1 μM) stabilizes K_v_1.5 channel protein in the endoplasmic reticulum and Golgi apparatus thereby enhancing the K_v_1.5 channel current on the cell membrane (Koshida et al., 2009).

As mentioned above, K_v_1.5 inhibition was observed when they were added to the external side of the membrane. This fact strongly suggests an interaction between *n*−3 PUFAs and ion channel, unlike the non-specific lipid-protein interactions proposed for marine *n*−3 PUFAs (Leaf and Xiao, 2001). This hypothesis was tested by measuring changes in the fluorescence anisotropy of the lipophilic dye PA-DPH on the membrane of *Ltk*^−^ cells in the presence of ALA or DHA. The PA-DPH molecule has an anion polar head and a lipid hydrocarbon chain that resembles membrane lipids. It is incorporated and anchored into the bilayer of the plasmalemma and its motion reflects the wobbling of its lipidic components. It was found that DHA but not ALA, modifies the PA-DPH motion in the *Ltk*^−^ cell membrane. From these experiments it was concluded that the apparent viscosity and the order of the membrane rather than the mobility of the bilayer components are not affected by ALA, but increased in cells incubated with DHA (Leifert et al., 1999; Guizy et al., 2008). These results do not permit us to exclude an effect of DHA on the biophysics of the lipid bilayer, besides its direct effects on ion channels. In fact, it has also been reported that *n*−3 PUFAs are able to change the composition of membrane microdomains (Basiouni et al., 2012; Turk and Chapkin, 2012).

The ultra rapid activating delayed rectifier potassium current *I*_Kur_, together with *I*_to_, repolarizes the cardiomyocyte membrane in human atria during the AP (Wang et al., 1993) but does not play a role in the ventricle even though its mRNA and protein are also present in that tissue (Mays et al., 1995). Thus, inhibition of *I*_Kur_ prolongs atrial but not ventricular APD and refractoriness. This finding indicates that *I*_Kur_ blockers can be useful in the treatment of supraventricular arrhythmias such as AF without the risk of ventricular pro-arrhythmia (Tamargo et al., 2004). Some of the cardioprotective effects attributed to *n*−3 PUFAs may be mediated by the inhibition of K_v_1.5 current. This could be particularly relevant on the treatment of AF. This arrhythmia is associated with rapid shortening of APD and ERP. In fact, recent epidemiological studies points out an important role of *n*−3 PUFAs in the prevention of AF after coronary by-pass surgery (Calo et al., 2005; Mariscalco et al., 2010).

While atrial repolarization is mostly carried out by *I*_to_ and *I*_Kur_, ventricular repolarization is developed by the slowly activating delayed rectifier (*I*_Ks_) and the rapidly activating delayed rectifier (*I*_Kr_) currents. The *I*_Kr_ is the native counterpart of K_v_11.1 channels present in the human myocardium (Sanguinetti and Jurkiewicz, 1991; Sanguinetti et al., 1995) and its activity determines the duration of the QT interval of the electrocardiogram (ECG) and therefore the refractory period (Li et al., 1996).

In 2002 it was demonstrated, for the first time, the inhibitory effects of DHA on *I*_K_ current (composed by *I*_Ks_ and *I*_Kr_) in ferret myocytes. The extracellular application of DHA (10 μM) inhibited *I*_K_ on atrial and ventricular ferret myocytes with an IC_50_ of ∼20 μM. K_v_11.1 blockade was concentration- but not voltage-dependent. Other PUFAs like the *n*−3 ALA and the *n*−6 linoleic acid also decreases the *I*_K_ magnitude whereas monounsaturated (OA) and saturated fatty acids (SA) did not (Xiao et al., 2002).

The same *n*−3 PUFA concentration (10 μM) was tested in another work to characterize the effects of DHA and AA on K_v_11.1 human cloned channels (Guizy et al., 2005). Both, AA and DHA reduced the K_v_11.1 current at the end of long depolarizing pulses and, to a greater extent, at the peak of the deactivation tail current, DHA being more potent. Also, K_v_11.1 block was voltage-, time-, and use-dependent. AA and DHA: (1) shifted the activation curve toward more negative membrane potentials (−5 and −11 mV, respectively) and shifted the inactivation curve toward more positive potentials (∼12 mV); (2) accelerated the activation and the deactivation kinetics; (3) at high rate frequencies, the K_v_11.1 peak current was exponentially reduced; and (4) did not modify the onset, the inactivation kinetics nor the recovery process. All these results suggest that AA and DHA preferentially bind to the open state of the channel (Guizy et al., 2005).

The effects of incorporated *n*−3 PUFAs were also evaluated on pig ventricular myocytes fed with a diet rich in *n*−3 PUFAs for 8 weeks. In this study, no changes on the amplitude and the properties of *I*_Kr_ were noticed, thus suggesting different effects of *n*−3 PUFAs after acute exposition than after feeding animals (i.e., chronic conditions; Verkerk et al., 2006).

*n*−3 PUFAs, like most antiarrhythmic drugs, modulate sodium, potassium, and calcium channels, as well as α- and β-adrenoceptors (Honoré et al., 1994; Xiao et al., 1995, 1997; Hondeghem et al., 2001; Jude et al., 2003; Guizy et al., 2005). EPA and DHA block sodium, calcium, and potassium currents with the exception of *I*_Ks_ and *I*_K1_ (Xiao et al., 1995, 1997; Doolan et al., 2002; Verkerk et al., 2006). Since *n*−3 PUFAs inhibit *I*_Na_, *I*_Ca_, and enhance *I*_Ks_ (effects that would shorten the cardiac action potential), but also inhibit *I*_to_, *I*_Kur_, and *I*_Kr_ (that should produce a lengthening of the APD), the result should be a modest effect on the time of repolarization. Besides these effects on the APD, it should be stated that its inhibitory effects on sodium, calcium, and several potassium channels (K_v_4, and K_v_11.1 channels) would result in a lengthening of the refractory period and a decrease of cardiac excitability, thus contributing to its antiarrhythmic effects.

The clinical consequences of the effects produced by *n*−3 PUFAs on K_v_11.1 channels are likely dependent on the underlying pathology; but we cannot state any conclusion from the data available in the literature.

## Effects of *n*−3 PUFAs on Arrhythmias Animal Models

In addition to the studies concerning the role of *n*−3 PUFAs on ion channels, several cellular, and animal models of induced arrhythmia have been created to better understand the mechanism by which *n*−3 PUFAs protect or exacerbate certain arrhythmias (Rosen et al., 1991; Members of the Sicilian Gambit, 2001). Traditional antiarrhythmic drug trials have yielded disappointing results, probably due to the inclusion of heterogeneous patient populations with different arrhythmogenic substrate. This led to the international cardiology community to focus their interest on the design of new rational approaches to study the effects of antiarrhythmic drugs based on mechanism- and disease-specific fashion. So, during the last years, the pro- or antiarrhythmic effects of *n*−3 PUFAs have been studied on different animal models with distinct underlying mechanisms of arrhythmia (triggered activity or reentry).

### Animal models based on arrhythmias produced by triggered activity

The effects of EPA and DHA upon APD, triangulation, reverse use dependence, instability and dispersion (TRIaD) were studied in female rabbit fed with food enriched with *n*−3 PUFA (Dujardin et al., 2008). In this study, *n*−3 PUFAs significantly prolonged APD and ERP without triangulation. This apparent discrepancy with another studies (Verkerk et al., 2006; Den Ruijter et al., 2010) can be related to the fact that this study was performed in female rabbit hearts, whose repolarization is known to be slower and is modulated in a different manner (Gaborit et al., 2010; Lowe et al., 2012). This prolongation of APD resulted in a significant prolongation of the ERP. At baseline, *n*−3 PUFAs pre-treatment had no effects on TRIaD and on ectopic activity compared with control hearts. By exposing the rabbit heart to increasing dofetilide concentrations (1–100 nM) torsades de pointes (TdP, polymorphic ventricular arrhythmia due to triggered activity) was induced. It was studied whether the *n*−3 PUFA pre-treatment could prevent the proarrhythmic actions of dofetilide by decreasing TRIaD, and by suppressing the appearance of TdP. In control hearts, dofetilide-induced TdP in five of the six rabbit hearts, but none in the hearts from the *n*−3 PUFAs group. Hence, dofetilide-induced TRIaD was attenuated by pre-treatment with dietary supplements of *n*−3 PUFAs (Dujardin et al., 2008). Thus, a diet rich in *n*−3 PUFAs reduce the incidence of early afterdepolarizations (EADs) that could be also benefit in the treatment of long QT syndrome and other arrhythmias developed by triggered activity (e.g., EADs; Den Ruijter et al., 2006; Dujardin et al., 2008).

It has been described the beneficial effects of *n*−3 PUFAs in arrhythmias associated to heart failure. These beneficial effects have been reported when *n*−3 PUFAs are administrated acutely or as a diet supplementation. The acute administration of fish oil in cardiac myocytes from rabbits with volume- and pressure-overload-induced heart failure and of patients with end-stage heart failure inhibited triggered arrhythmias by lowering intracellular calcium and decreasing the response to noradrenaline (Den Ruijter et al., 2008). In this study, it is also reported a shortening of the cardiac APD induced by *n*−3 PUFAs. Recently, in the same animal model the authors demonstrate that supplementation of the diet with *n*−3 PUFAs reduces hypertrophy. They also describe a shortening of the cardiac action potential due to an increase in the magnitude of *I*_to_ and *I*_K1_, a decrease of the Ca^2+^ transients and of the incidence of delayed afterdepolarizations. Thus, *n*−3 PUFAs prevented electric remodeling associated with heart failure, leading to a reduced susceptibility of arrhythmias (Den Ruijter et al., 2012).

### Animal models based on reentrant arrhythmias

Dietary *n*−3 fatty acids decreased the incidence of fatal heart disease relative to placebo (GISSI-Prevenzione Investigators, 1999). However, *n*−3 PUFAs tended to increase the risk of SCD in patients with angina pectoris (without myocardial infarction; Burr et al., 2003). Coronel et al. (2007) demonstrated in a pig ischemia model (produced by occluding the left anterior descending artery) that the supplementation of the diet with *n*−3 PUFAs during 8 weeks, did not modified ERP or the conduction velocity (neither longitudinal or transversal), although increased the incidence of arrhythmias. This study reports that those animals fed with *n*−3 PUFAs enriched diet show a reduced excitability, likely due to their effects on sodium channels, the main determinant of the substrate of ischemia-induced reentrant arrhythmias.

## New Insights of the Beneficial PUFAs Properties: PUFAs Metabolites Effects

In addition, genetic, pharmacologic, and biochemical studies are providing increasing mechanistically relevant explanations for the salutatory actions of EPA and DHA. *n*−3 and *n*−6 PUFAs are metabolized to lipoxins, resolvins, maresins (Serhan et al., 2008; Bannenberg and Serhan, 2010). These EPA- and DHA-derived lipid mediators (collectively termed Specific Pro-resolving Mediators, SPMs) are active, as anti-inflammatory agents, at very low concentrations (picomolar to nanomolar range), and are degraded locally by specific inactivating enzymes, characteristic for the bioactivity of autacoids. These SPMs can also slow the progression of diabetic onset, CVD, and aging-associated pathologies through the regulation of innate and adaptive immune responses. It has been described that lipoxins modulate CRAC channels after binding to the lipoxin receptor (Li et al., 2008). The effects of SPMs in the progression of several CVDs, involving inflammation (i.e., heart failure, diabetes, coronary heart disease) can be associated to the beneficial observed effects with *n*−3 PUFAs in the clinical trials. However, the cardiac electrophysiological effects of these SPMs are unknown at the present time. Thus, further studies focused on the analysis of their effects on cardiac ion channels and arrhythmias are necessary to better understanding the pro- or antiarrhythmic effects of *n*−3 PUFAs.

## Concluding Remarks

*n*−3 PUFAs have diverse effects (increasing and decreasing) on cardiac ion currents. These differences appeared to depend on whether the *n*−3 PUFAs were applied to the cells or incorporated in the membranes modifying its composition and, thus, the effects on cell signaling and, therefore, in ion channel function. However, we cannot rule out PUFAs direct effects on ion channels, since once incorporated, *n*−3 PUFAs can also be released and there will arise an equilibrium between incorporated and circulating. *n*−3 PUFAs modulate cardiac ion currents, leading to a shortening of the cardiac APD. This effect can be beneficial or harmful, depending on the underlying pathology. Thus, during acute ischemia, in which the duration of the cardiac APD is already shortened (Behrens et al., 1997), a further decrease should be proarrhythmic. However, the APD shortening should be beneficial in preventing those arrhythmias caused by triggered activity. These differences can account, at least partially, with discrepancies observed in the clinical and epidemiological studies. Future studies analyzing the electrophysiological effects on cardiac ion channels and different models of arrhythmia of the *n*−3 metabolites are needed to better understanding the action of *n*−3 PUFAs.

## Conflict of Interest Statement

The authors declare that the research was conducted in the absence of any commercial or financial relationships that could be construed as a potential conflict of interest.
